# Supplementary material for: Structural connectome architecture shapes the maturation of cortical morphology from childhood to adolescence
Source: Nat Commun. 2024 Jan 26;15:784. doi: 10.1038/s41467-024-44863-6 (PMC10817914; doi:10.1038/s41467-024-44863-6)
Supplement: Supplementary file 1 — Supplementary Information [file 41467_2024_44863_MOESM1_ESM.pdf]

Supplementary Information (SI) for

Structural connectome architecture shapes the maturation of cortical morphology from childhood to adolescence

Xinyuan Liang, Lianglong Sun, Xuhong Liao, Tianyuan Lei, Mingrui Xia, Dingna Duan, Zilong Zeng, Qiongling Li, Zhilei Xu, Weiwei Men, Yanpei Wang, Shuping Tan, Jia-Hong Gao, Shaozheng Qin, Sha Tao, Qi Dong, Tengda Zhao, Yong He

Corresponding authors:

Yong He, Ph.D., E-mail: yong.he@bnu.edu.cn
Tengda Zhao, Ph.D., E-mail: tengdazhao@bnu.edu.cn

SI-1. Participants and MRI Data Analyses

1.1 Participants

We performed analyses in two independent datasets. The discovery dataset included a longitudinal cohort of 358 typically developing participants (aged 6-14 years, 651 scans from 195 males and 163 females) from the Beijing Cohort in Children Brain Development (CBD) project. All the individuals were recruited from primary schools in Beijing. The exclusion criteria included cognitive anomalies [1], significant physical illness or history of neurological/psychiatric disorders, significant head injuries, abuse of illegal drugs and alcohol, or contraindications for MRI. Some of these participants were invited to take part in up to three repeated MRI scans, with an interval of approximately one year between each scan. Informed written consent was obtained from all participants and at least one parent/guardian, consistent with the guidelines of the Ethics Committee of Beijing Normal University.

After strict quality control, a total of 130 scans were excluded. Specifically, 76 scans were excluded due to artifacts in T1-weighted (T1w) images, 39 and 15 scans were excluded due to high in-scanner motion (maximum head motion > 3 mm) and serious signal dropout, respectively in diffusion MRI (dMRI) images. Finally, 521 scans from 314 participants (aged 6-14 years, 153 females) were included in the study.

For reproducibility analyses, we also included a cross-sectional replication dataset from the Lifespan Human Connectome Project in Development (HCP-D) [2]. Participants were recruited across four imaging sites: (1) Harvard University, (2) University of California-Los Angeles, (3) University of Minnesota, and (4) Washington University in St. Louis (WUSTL). Details on the inclusion and exclusion criteria can be found in [2]. All procedures were approved by a central Institutional Review Board administered at Washington University in St. Louis (IRB #201603135).

In this replication dataset, we initially included 301 typically developing participants aged 5 to 14 years (183 females). First, we excluded 3 participants with anatomical anomalies and 17 participants with notable myelin map quality issues according to each subject's quality control report of structural MR images [2]. After these, T1w images of 98 children (5-10 years, mean age 8.72 ± 0.99 years, 32 males) and 203 adolescents (10-14 years, mean age 12.17 ± 1.28 years, 86 males) were eventually included. Next, for dMRI images, we excluded 30 participants due to serious signal dropout (based on eddy-corrected dMRI images) and 4 participants due to high in-scanner motion (maximum head motion > 3 mm) in the child group. After these, diffusion images from 64 children were included in this study.

1.2 Image Acquisition

For the discovery dataset, high-resolution T1w images for each subject were scanned at Peking University using a 3T Siemens Prisma scanner. T1w images were acquired using the following parameters: repetition time (TR) = 2530 ms, echo time (TE) = 2.98 ms, inversion time (TI) = 1100 ms, flip angle (FA) =7°, acquisition matrix = 256×224, field of view (FOV) = 256×224 mm^2, slice number = 192, in-plane resolution = 1.0 × 1.0 mm, slice thickness = 1 mm, bandwidth (BW) = 240 Hz/Px. Diffusion-weighted images were acquired using the high angular resolution diffusion imaging (HARDI) sequence with a 64-channel head coil with parameters as follows:

TR = 7500 ms, TE = 64 ms, acquisition matrix = 112×112, FOV = 224×224 mm^2, slices = 70, in-plane resolution = 2.0 × 2.0 mm, slice thickness = 2.0 mm, BW = 2030 Hz/Px, phase encoding = P->A, 64 diffusion weighted directions (b-value = 1000 s/mm^2) with 10 non-diffusion weighted b0 (0 s/mm^2). Meanwhile, additional fieldmap images were obtained for EPI distortion correction with the following parameters: acquisition matrix = 112×112, FOV =224×224 mm^2, slices = 70, slice thickness = 2.0 mm, TR = 695 ms, TE1 = 4.92 ms, TE2 = 7.38 ms, in-plane resolution = 2.0 × 2.0 mm.

For the replication dataset, high-resolution T1w images were scanned on a 3T Siemens Prisma using the following parameters [3]: TR = 2500 ms, TE = 1.8/3.6/5.4/7.2 ms, TI = 1000 ms, FA = 8°, in-plane resolution = 0.8 × 0.8 mm, slice thickness = 0.8 mm, and in-plane acceleration factor = 2. Parameters for diffusion MRI were as follows: TR = 3230 ms, TE = 89 ms, in-plane resolution = 1.5 × 1.5 mm, slice thickness = 1.5 mm, multiband acceleration factor = 4, 92-93 directions per shell (b = 1500/3000 mm^2). There are 28 b0 volumes equally interspersed across four consecutive dMRI runs. Therefore, each individual acquired two images with 199 volumes in the opposite phase encoding direction (AP and PA).

SI-2. Estimating the Maturation Pattern of CT and Its Associations with WM Connectome Structure from Childhood to Adolescence

2.1 WM Connectome Construction

We reconstruct anatomical streamlines between each two cortical regions based on diffusion MR images to generate the cortical WM network. This procedure was constructed using DSI Studio software (https://www. nitrc.org/projects/dsistudio). First, an SRC file was generated from the dMRI image for each child. Then, we generated spin distribution function (SDF) maps using the generalized q-sampling imaging (GQI) algorithm [4] with a diffusion sampling length ratio of 1.25. The GQI is a model-free method to estimate the anisotropy of diffusing water, which is suitable for reconstructing crossing fibers [5]. Then, the deterministic fiber tracking [6] was performed in the individual native dMRI space according to the following steps: 1) We first generated the gray-white boundary mask by dilating the gray matter (GM) atlas 2 voxels toward the inner boundaries and taking the intersection of the WM mask; 2) We merged the original GM atlas and the GM-WM boundary to obtain a seed mask and defined the terminative mask as cerebrospinal fluid (CSF) and subcortical regions; 3) All these masks were transformed from the native T1w space into the native dMRI space using the individual mean b0 image as the co-registered target in ANTS [7]; 5) Ten million streamlines were generated by using the whole individual dMRI images, the transformed seed mask and the transformed terminative mask as inputs and with a step size of 0.625 mm. The anisotropy threshold was set as default, and the turning angle threshold was 45°. Streamlines smaller than 6 mm or larger than 250 mm were further removed. Next, we obtain the individual WM network by defining two cortical regions structurally connected if there exists at least one streamline with two end-points located separately in them [8]. To ensure that the WM network is fully connected, we did not perform additional thresholding operations in this study. For each participant, the connectivity matrices weighted by streamline density (defined as the fiber number normalized by the mean surface area of the two brain regions) were generated using the same parcellations as described above. Finally, the binary group-level WM backbones were created using a consensus approach that preserves the connection length distributions of individual children [9].

2.2 Association between nodal attributes of the WM network and CT maturation

To seek more direct evidence of an association between the WM network topology and cortical maturation, we conducted a new analysis to quantify the correlation between nodal graph-theoretical attributes of the WM network and nodal CT maturation (obtained from Statistical Model I). We selected three nodal topological metrics to measure the regional capacity of information transfer in common communication dynamics [10, 11], including nodal efficiency (Eff), the nodal mean first passage time (MFPT), and the nodal participation coefficient (PC). Nodal Eff is the normalized sum of the reciprocal of shortest path lengths from a given node to all other brain nodes and represents the ability to transfer information through routing communications. Nodal MFPT quantifies the average time taken to move from one node to all other nodes for the first visit through a random walk. It reflects the ability to transfer information through diffusion-based paths rather than prior selected paths in routing communications. A smaller nodal MFPT reflects higher diffusive efficiency. The nodal PC measures the ratio of a node's between-community connections to its total connections. Nodes with high PC values indicate strong information transfer abilities during network integration. To obtain an overall view of the entire developmental period, we utilized the differences in nodal CT identified in group comparisons (children vs. adolescents, Statistical Model I) as indicators of the nodal cortical maturation extent.

SI-3. Relationship between Heterogeneous Connectome Constraints on Cortical Maturation and Gene Expression Profiles

3.1 BrainSpan Atlas

The BrainSpan data [12] provided developmental gene expression in brain tissue samples from 8 postconception weeks to 40 years. The 42 donors were divided into five groups based on their age, including fetal (8-37 postconception weeks), infant (4 months-1 year), child (2-8 years), adolescent (11-19 years), and adult (21-40 years). Only neocortical regions were included in our study. There were four tissue samples excluded (primary motor-sensory cortex (samples), occipital neocortex, parietal neocortex, temporal neocortex) with gene expression values only in the early fetal period [13]. Genes with 0 expression values in all tissue samples were removed. Then, we divided these samples into dominant and non-dominant categories according to their anatomical location (from 11 areas of the neocortex) and arranged them in ascending order based on age to explore the temporal characteristics of gene expression. Next, we selected four gene sets [14] that cover typical maturation procedures involved in both CT and WM, including axon development, myelination, dendrite development, and synapse development, to evaluate whether there are differences in transcription levels between dominant and non-dominant regions. For each gene set, we performed principal component analysis (PCA) on the gene expression matrix to calculate the first principal component score of each gene set’s transcription level in dominant and non-dominant regions. The transcriptomic trajectories were characterized by using locally weighted regression to fit the first principal component score with the postconceptional days (log2) as in a previous study [14]. For visualization, we scaled the first principal component score to the range 0-1 across all tissue samples using min-max normalization.

To assess the significance of the difference for each gene set, we calculated the difference between the means of the first principal component scores of the two categories of brain regions. Please note that we only include tissue samples within the age range of individuals included in

the CBD dataset (6 to 14 years) here. Specifically, there were 16 sample tissues (mean age 10 ± 2.19 years) in dominant regions and 25 sample tissues (mean age 10.24 ± 2.17 years) in non-dominant regions. Of note, these two groups of samples were matched in age distribution (*t*-value = -0.34, P = 0.73). Next, we randomly sampled an equal number of genes with each gene set from the remaining genes in BrainSpan datasets and recalculated the difference and compared the observed transcription level differences against the null distributions generated by repeating 1000 permutation tests [15, 16, 17].

3.2 Allen Human Brain Atlas

The regional gene expression data were obtained from Allen Human Brain Atlas datasets (http://human.brain-map.org) [18]. Since only two of the six donors (mean age: 42.50 ± 13.38 years; 1 female) contained whole-brain data, we only considered the left hemisphere here. To obtain the gene transcriptional profile of each brain region, the regional microarray expression data were preprocessed using a recommended pipeline with the abagen toolbox [19, 20]. First, probe reannotation was performed according to the information provided by [19]. Second, we used the intensity-based method to remove the probes that did not exceed background noise in at least 50% of tissue samples across all donors. When multiple probes can represent the expression of the same gene, we retained the probe with the highest differential stability across donors. The differential stability was defined as:

$$DS(p) = \frac{1}{\binom{N}{2}} \sum_{i=1}^{N-1} \sum_{j=i+1}^{N} r\left[B_i(p), B_j(p)\right] \tag{1}$$

where N is the number of donors, p is a single probe, and r is the Spearman correlation of microarray expression values across brain regions in donor B_i and donor B_j. Next, the MNI coordinates of the samples were updated to those generated by using nonlinear registration. Under the guidance of MNI coordinates, tissue samples were assigned to the brain regions according to the given atlas by searching the nearest brain region within 2 mm. In this process, hemisphere and gross structural information were also used to reduce the assigning bias. All samples not assigned to a brain region were discarded.

To mitigate the differences in microarray expression between donors, we used a scaled robust sigmoid function to normalize each sample across all genes [21].

$$x_{norm} = \frac{1}{1 + exp\left(\frac{-(x_i - \langle x \rangle)}{IQR_x}\right)} \tag{2}$$

where $\langle x \rangle$ represents the median and IQR is the interquartile range. Then, we rescaled the normalized expression values to the unit interval using the min-max function:

$$x_{scaled} = \frac{x_{norm} - min(x_{norm})}{max(x_{norm}) - min(x_{norm})} \tag{3}$$

Each gene expression value was normalized across all samples by using the same method. Tissue samples assigned to the same parcel were averaged separately for each donor and then averaged

across six donors. Finally, stable genes with differential stability greater than 0.1 were retained for our analysis [13]. Genes with greater differential stability are more consistent across donors and more biologically relevant, such as disease, drug targets, and literature citations [18]. After preprocessing, a gene expression matrix (111 brain regions × 8631 gene expression levels) was generated. Then, we identified the association between the dominant likelihood map at the 3rd neighboring scale (which exhibited the highest prediction accuracy) and each gene expression map using Pearson’s correlation and spin tests (1000 times).

3.3 Gene Ontology Enrichment Analysis

We performed the Gene Ontology enrichment analysis on gene sets using the ToppGene Suite [22] according to the following thresholds: (1) *P* value cutoff was 10^{-5} in the advanced parameter settings, (2) *q-value* < 0.05 in Benjamini–Hochberg false discovery rate (FDR) corrections. The significant enrichment terms for both positively correlated and negatively correlated gene sets are shown in Tables S6-S7. We used the online tool REViGO (http://revigo.irb.hr) to select the most meaningful GO terms.

Table S1 Accuracy of the model at predicting the spatial maturation of CT by using multiscale diffusion profiles of network links as features (1000-node resolution).

Neighboring scale	Accuracy	$p_{rewired}$	p_{spin}
1	0.74	<0.001	<0.001
2	0.74	<0.001	<0.001
3	0.75	<0.001	<0.001
4	0.73	<0.001	<0.001
5	0.71	<0.001	<0.001
6	0.69	<0.001	0.001
7	0.67	<0.001	0.001
8	0.66	<0.001	0.001
9	0.65	<0.001	0.001

Note: The table above shows the prediction accuracies and *p*-values (calculated as the fraction of null values exceeding the observed accuracy in the "rewired" test and "spin" test, one-sided) yielded by an SVR model with multiscale diffusion profiles of WM network links as features to predict the spatial maturation of CT (obtained from Statistical Model I). Spatial maturation of CT and diffusion profiles of WM network links were obtained from CBD dataset.

Table S2 Accuracy of the model at predicting the spatial maturation of CT by using multiscale diffusion profiles of network links as features (219-node resolution).

Neighboring scale	Accuracy	$p_{rewired}$	p_{spin}
1	0.64	<0.001	0.001
2	0.64	<0.001	0.001
3	0.65	<0.001	<0.001
4	0.61	<0.001	0.001
5	0.60	<0.001	<0.001
6	0.56	<0.001	0.001

Note: The table above shows the prediction accuracies and *p*-values (calculated as the fraction of null values exceeding the observed accuracy in the "rewired" test and "spin" test, one-sided) yielded by an SVR model with multiscale diffusion profiles of WM network links as features to predict the spatial maturation of CT (obtained from Statistical Model I). Spatial maturation of CT and diffusion profiles of WM network links were obtained from CBD dataset.

Table S3 Accuracy of the model at predicting the spatial maturation of CT by using multiscale diffusion profiles of network links as features (448-node resolution).

Neighboring scale	Accuracy	$p_{rewired}$	p_{spin}
1	0.72	<0.001	<0.001
2	0.73	<0.001	<0.001
3	0.73	<0.001	<0.001
4	0.72	<0.001	<0.001
5	0.69	<0.001	<0.001
6	0.68	<0.001	<0.001
7	0.68	<0.001	<0.001

Note: The table above shows the prediction accuracies and *p*-values (calculated as the fraction of null values exceeding the observed accuracy in the "rewired" test and "spin" test, one-sided) yielded by an SVR model with multiscale diffusion profiles of WM network links as features to predict the spatial maturation of CT (obtained from Statistical Model I). Spatial maturation of CT and diffusion profiles of WM network links were obtained from CBD dataset.

Table S4. Accuracy of the model at predicting the rates of CT maturation at each age by using multiscale diffusion profiles of network links as features.

		Neighboring scale								
		**1**	**2**	**3**	**4**	**5**	**6**	**7**	**8**	**9**
**6 y**	r	0.68	0.68	0.68	0.66	0.65	0.64	0.62	0.61	0.60
	p_{spin}	<0.001	<0.001	<0.001	<0.001	<0.001	<0.001	0.001	0.003	0.006
	$p_{rewired}$	all < 0.001								
**8 y**	r	0.68	0.68	0.68	0.67	0.66	0.64	0.63	0.62	0.60
	p_{spin}	<0.001	<0.001	<0.001	<0.001	<0.001	<0.001	0.001	0.002	0.004
	$p_{rewired}$	all < 0.001								
**10 y**	r	0.70	0.70	0.71	0.68	0.66	0.65	0.63	0.62	0.61
	p_{spin}	<0.001	<0.001	<0.001	<0.001	<0.001	<0.001	<0.001	0.001	0.002
	$p_{rewired}$	all < 0.001								
**12 y**	r	0.64	0.62	0.62	0.60	0.59	0.59	0.57	0.56	0.55
	p_{spin}	all < 0.001								
	$p_{rewired}$	all < 0.001								
**14 y**	r	0.63	0.62	0.62	0.59	0.58	0.58	0.56	0.55	0.55
	p_{spin}	all < 0.001								
	$p_{rewired}$	all < 0.001								

Note: The table above shows the prediction accuracies and p-values (calculated as the fraction of null values exceeding the observed accuracy in the "rewired" test and "spin" test, one-sided) yielded by an SVR model with multiscale diffusion profiles of WM network links as features to predict the CT maturation rates at each age (obtained from Statistical Model II, GAM analysis). Maturation rates of nodal CT and diffusion profiles of WM network links were obtained from the CBD dataset.

Table S5 List of genes showing the highest positive and negative correlations with the dominant likelihood map in the main analysis.

	Genes	Correlation	p_{spin}
Positive	NPHP1	0.66	< 0.001
	FAM133A	0.64	< 0.001
	PPFIA2	0.63	< 0.001
	FAM185A	0.63	< 0.001
	BCL2	0.63	< 0.001
	EID2B	0.62	< 0.001
	CHSY3	0.62	< 0.001
	GRM5	0.62	< 0.001
	CCDC120	0.62	< 0.001
	MLIP	0.62	< 0.001
Negative	SNX24	-0.65	< 0.001
	GNA14	-0.64	< 0.001
	NEFL	-0.64	< 0.001
	SLC7A1	-0.62	< 0.001
	DLC1	-0.61	< 0.001
	GBE1	-0.61	< 0.001
	FAM189B	-0.60	< 0.001
	KANK4	-0.60	< 0.001
	TRADD	-0.60	< 0.001
	DPY19L1	-0.60	0.003

Note: The table above gives the top 10 genes most positively and negatively associated with the dominant likelihood map, respectively. One-sided *p*-values were calculated. The detailed analysis results for all genes are available at https://github.com/Xinyuan-Liang/SC-shapes-the-maturation-of-cortical-morphology/releases/tag/v1.0.0.

Table S6 Enrichment analysis (GO terms of biological processes and cellular component) reveals significant positive correlations with the dominant likelihood map in the main analysis.

	GO term ID	GO term	Raw *p*-value	*q*_value FDR
Biological Processes	GO:0007611	learning or memory	4.38E-06	1.16E-02
	GO:0050808	synapse organization	6.77E-06	1.16E-02
	GO:0048666	neuron development	9.14E-06	1.16E-02
	GO:0050890	cognition	9.62E-06	1.16E-02
Cellular Component	GO:0098978	glutamatergic synapse	1.22E-08	7.34E-06
	GO:0097060	synaptic membrane	3.55E-08	1.07E-05
	GO:0098794	postsynapse	1.29E-07	2.57E-05
	GO:0045211	postsynaptic membrane	1.71E-07	2.57E-05
	GO:0099240	intrinsic component of synaptic membrane	3.40E-07	4.10E-05
	GO:0043197	dendritic spine	4.80E-07	4.83E-05
	GO:0044309	neuron spine	6.20E-07	5.34E-05
	GO:0098936	intrinsic component of postsynaptic membrane	1.67E-06	1.24E-04
	GO:0099699	integral component of synaptic membrane	1.86E-06	1.24E-04
	GO:0030425	dendrite	2.23E-06	1.29E-04
	GO:0097447	dendritic tree	2.36E-06	1.29E-04
	GO:0032279	asymmetric synapse	5.76E-06	2.90E-04
	GO:0043005	neuron projection	6.72E-06	3.12E-04
	GO:0045202	synapse	7.90E-06	3.40E-04
	GO:0036477	somatodendritic compartment	9.77E-06	3.93E-04

Note: The table above gives the significant enrichment terms for genes that show significant positive correlations with the dominant likelihood map. One-sided *p*-values were calculated. We used the online tool REViGO (http://revigo.irb.hr) to select the most meaningful GO terms and highlight them. The detailed enrichment analysis results for all genes are available at https://github.com/Xinyuan-Liang/SC-shapes-the-maturation-of-cortical-morphology/releases/tag/v1.0.0.

Table S7 Enrichment analysis (GO terms of biological processes and cellular component) reveals significant negative correlations with the dominant likelihood map in the main analysis.

	GO term ID	GO term	Raw *p*-value	*q*_value FDR
Biological Processes	GO:0019752	carboxylic acid metabolic process	5.39E-10	3.19E-06
	GO:0043436	oxoacid metabolic process	1.56E-09	4.44E-06
	GO:0006082	organic acid metabolic process	2.25E-09	4.44E-06
	GO:0006091	generation of precursor metabolites and energy	3.49E-09	5.16E-06
	GO:0019637	organophosphate metabolic process	5.12E-09	6.05E-06
	GO:0006163	purine nucleotide metabolic process	2.00E-08	1.97E-05
	GO:0009117	nucleotide metabolic process	2.61E-08	2.21E-05
	GO:0006753	nucleoside phosphate metabolic process	3.55E-08	2.62E-05
	GO:0072521	purine-containing compound metabolic process	4.57E-08	3.00E-05
	GO:0045333	cellular respiration	6.86E-08	4.06E-05
	GO:0015980	energy derivation by oxidation of organic compounds	1.24E-07	6.69E-05
	GO:0072522	purine-containing compound biosynthetic process	2.78E-07	1.37E-04
	GO:0009144	purine nucleoside triphosphate metabolic process	5.38E-07	2.36E-04
	GO:0006164	purine nucleotide biosynthetic process	5.57E-07	2.36E-04
	GO:0055086	nucleobase-containing small molecule metabolic process	8.55E-07	3.05E-04
	GO:0009060	aerobic respiration	9.08E-07	3.05E-04
	GO:0009150	purine ribonucleotide metabolic process	9.12E-07	3.05E-04
	GO:1902600	proton transmembrane transport	9.27E-07	3.05E-04
	GO:0006811	ion transport	1.25E-06	3.66E-04
	GO:0009205	purine ribonucleoside triphosphate metabolic process	1.29E-06	3.66E-04
	GO:0019693	ribose phosphate metabolic process	1.30E-06	3.66E-04
	GO:0009152	purine ribonucleotide biosynthetic process	1.60E-06	4.30E-04
	GO:0009141	nucleoside triphosphate metabolic process	1.94E-06	4.72E-04
	GO:0009199	ribonucleoside triphosphate metabolic process	1.99E-06	4.72E-04
	GO:0046390	ribose phosphate biosynthetic process	1.99E-06	4.72E-04
	GO:0009259	ribonucleotide metabolic process	2.37E-06	5.38E-04
	GO:0046034	ATP metabolic process	2.70E-06	5.78E-04
	GO:0006812	cation transport	2.74E-06	5.78E-04
	GO:1901135	carbohydrate derivative metabolic process	3.07E-06	6.25E-04
	GO:0046434	organophosphate catabolic process	3.88E-06	7.65E-04
	GO:0009260	ribonucleotide biosynthetic process	4.46E-06	8.52E-04
	GO:0090407	organophosphate biosynthetic process	6.74E-06	1.21E-03
	GO:0034220	ion transmembrane transport	6.76E-06	1.21E-03
	GO:0022900	electron transport chain	7.56E-06	1.32E-03
	GO:0009165	nucleotide biosynthetic process	8.44E-06	1.43E-03
	GO:1901293	nucleoside phosphate biosynthetic process	8.88E-06	1.46E-03
	GO:0015986	proton motive force-driven ATP synthesis	9.25E-06	1.48E-03
Cellular Component	GO:0043209	myelin sheath	4.29E-16	3.20E-13
	GO:0005743	mitochondrial inner membrane	3.35E-11	1.05E-08
	GO:0019866	organelle inner membrane	4.24E-11	1.05E-08
	GO:0031966	mitochondrial membrane	5.64E-10	1.05E-07
	GO:0005740	mitochondrial envelope	2.07E-09	3.08E-07
	GO:0098798	mitochondrial protein-containing complex	3.16E-09	3.92E-07
	GO:1990204	oxidoreductase complex	3.15E-08	3.35E-06
	GO:0005759	mitochondrial matrix	1.24E-07	1.16E-05
	GO:0031967	organelle envelope	1.60E-07	1.19E-05
	GO:0031975	envelope	1.60E-07	1.19E-05
	GO:0098800	inner mitochondrial membrane protein complex	3.12E-06	2.12E-04

Note: The table above gives the significant enrichment terms for genes that show significant negative correlations with the dominant likelihood map (one-sided). We used the online tool REViGO (http://revigo.irb.hr) to select the most meaningful GO terms and highlight them. The detailed enrichment analysis results for all genes are available at https://github.com/Xinyuan-Liang/SC-shapes-the-maturation-of-cortical-morphology/releases/tag/v1.0.0.

Table S8 Accuracy of the model at predicting the spatial maturation of CT by using multiscale diffusion profiles of network links from HCP-D dataset as features (1000-node resolution).

Neighboring scale	Accuracy	$p_{rewired}$	p_{spin}
1	0.78	<0.001	<0.001
2	0.77	<0.001	<0.001
3	0.77	<0.001	<0.001
4	0.75	<0.001	<0.001
5	0.73	<0.001	<0.001
6	0.71	<0.001	<0.001
7	0.70	<0.001	<0.001
8	0.69	<0.001	<0.001

Note: The table above shows the prediction accuracies and *p*-values (calculated as the fraction of null values exceeding the observed accuracy in the "rewired" test and "spin" test, one-sided) yielded by an SVR model with multiscale diffusion profiles of WM network links as features to predict the spatial maturation of CT (obtained from Statistical Model I). The spatial maturation of CT was obtained from CBD dataset. Diffusion profiles of WM network links were obtained from HCP-D dataset.

Table S9 Accuracy of the model at predicting the spatial maturation of CT by using multiscale diffusion profiles of network links as features (1000-node resolution, independent tests in HCP-D dataset).

Neighboring scale	Accuracy	$p_{rewired}$	p_{spin}
1	0.66	<0.001	<0.001
2	0.65	<0.001	<0.001
3	0.65	<0.001	<0.001
4	0.60	<0.001	0.015
5	0.57	<0.001	0.152
6	0.55	<0.001	0.206
7	0.53	<0.001	0.313
8	0.51	<0.001	0.439

Note: The table above shows the prediction accuracies and *p*-values (calculated as the fraction of null values exceeding the observed accuracy in the "rewired" test and "spin" test, one-sided) yielded by an SVR model with multiscale diffusion profiles of WM network links as features to predict the spatial maturation of CT (obtained from Statistical Model I). Spatial maturation of CT and diffusion profiles of WM network links were both obtained from HCP-D dataset.

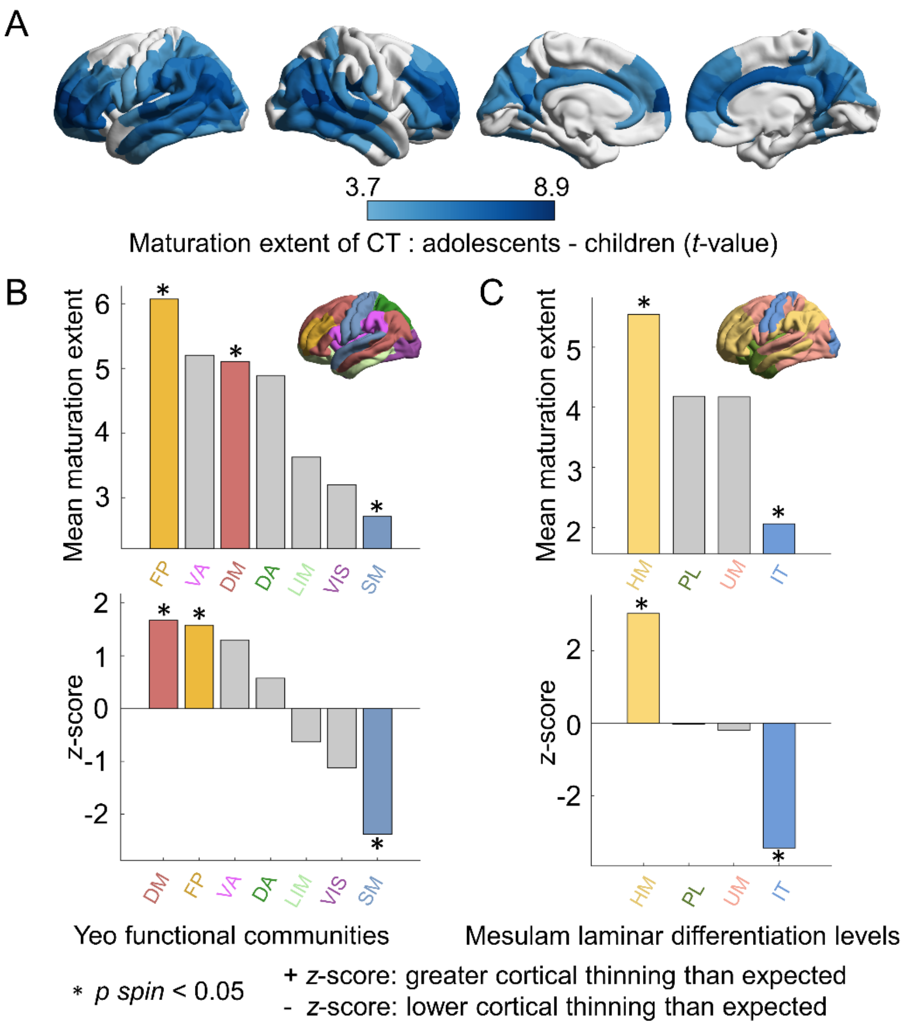

Figure S1. Validation of CT maturation from childhood to adolescence at 219-node resolution. (**A**) Spatial maturation map of CT (obtained from Statistical Model I) from childhood to adolescence. A greater positive *t*-value denotes more pronounced cortical thinning with development. The maps were corrected using a Bonferroni correction method for multiple comparisons ($p_{bonf} = 2.28\times10^{-4}$, two-sided). (**B**) The mean CT maturation extent (estimated by *t*-value) within each brain community was defined by Yeo et al. [23], and the laminar differentiation level was defined by Mesulam et al. [24] (**C**). Spin tests [25, 26] were performed by spherical projection and rotation class positions 1000 times for correcting spatial autocorrelations, and the class-specific mean *t*-values were expressed as *z* scores relative to this null model. A positive *z* score indicated higher cortical thinning than expected by chance. Asterisks denote statistical significance at $p_{spin} < 0.05$ (p_{spin}(FP) = 0.045, p_{spin}(DM) = 0.035, p_{spin}(SM) = 0.005, p_{spin}(HM) < 0.001, and p_{spin}(IT) = 0.001, one-sided). VIS, visual; SM, somatomotor; LIM, limbic; DA, dorsal attention; VA, ventral attention; FP, frontoparietal; DM, default mode; IT, idiotypic; PL, paralimbic; UM, unimodal and HM, heteromodal.

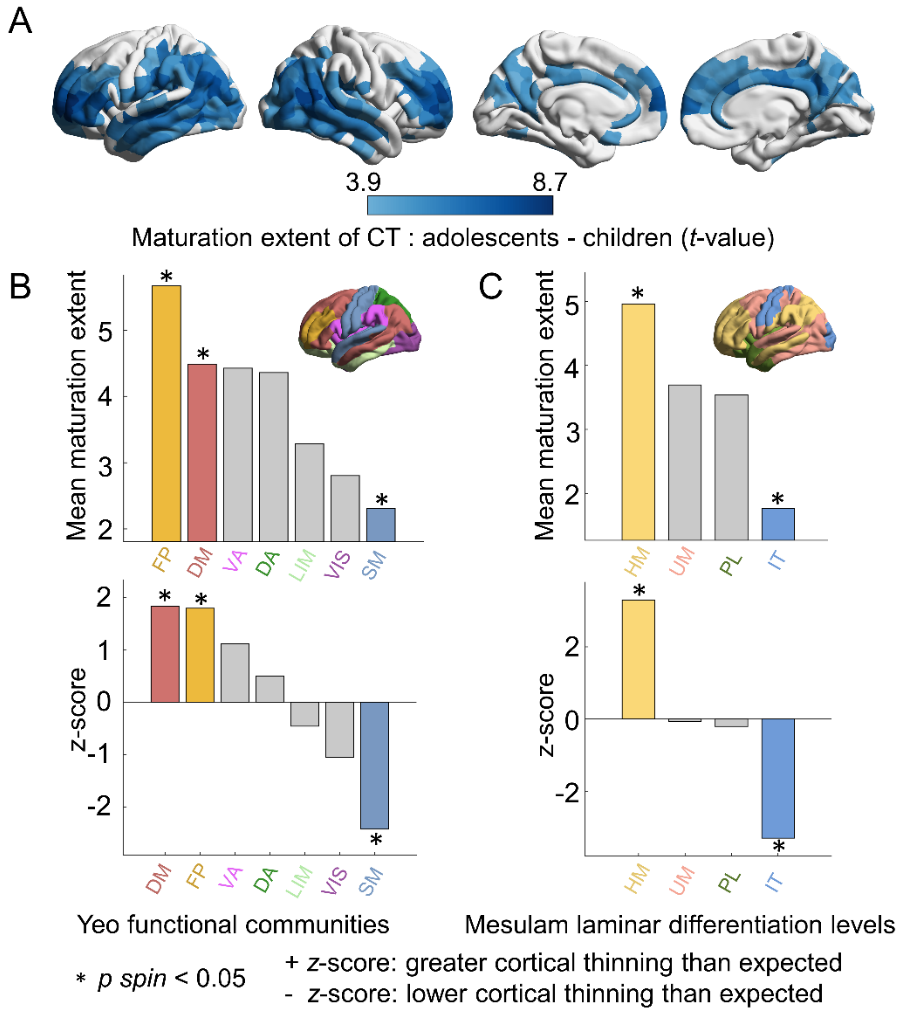

Figure S2. Validation of CT maturation from childhood to adolescence at 448-node resolution. (**A**) Spatial maturation map of CT (obtained from Statistical Model I) from childhood to adolescence. A greater positive *t*-value denotes more pronounced cortical thinning with development. The maps were corrected using a Bonferroni correction method for multiple comparisons ($p_{bonf} = 1.12\times10^{-4}$, two-sided). (**B**) The mean CT maturation extent (estimated by *t*-value) within each brain community was defined by Yeo et al. [23], and the laminar differentiation level was defined by Mesulam et al. [24] (**C**). Spin tests [25, 26] were performed by spherical projection and rotation class positions 1000 times for correcting spatial autocorrelations, and the class-specific mean *t*-values were expressed as *z* scores relative to this null model. A positive *z* score indicated higher cortical thinning than expected by chance. Asterisks denote statistical significance at $p_{spin} < 0.05$ (p_{spin}(FP) = 0.026, p_{spin}(DM) = 0.032, p_{spin}(SM) = 0.001, p_{spin}(HM) < 0.001, and p_{spin}(IT) = 0.001, one-sided). VIS, visual; SM, somatomotor; LIM, limbic; DA, dorsal attention; VA, ventral attention; FP, frontoparietal; DM, default mode; IT, idiotypic; PL, paralimbic; UM, unimodal and HM, heteromodal.

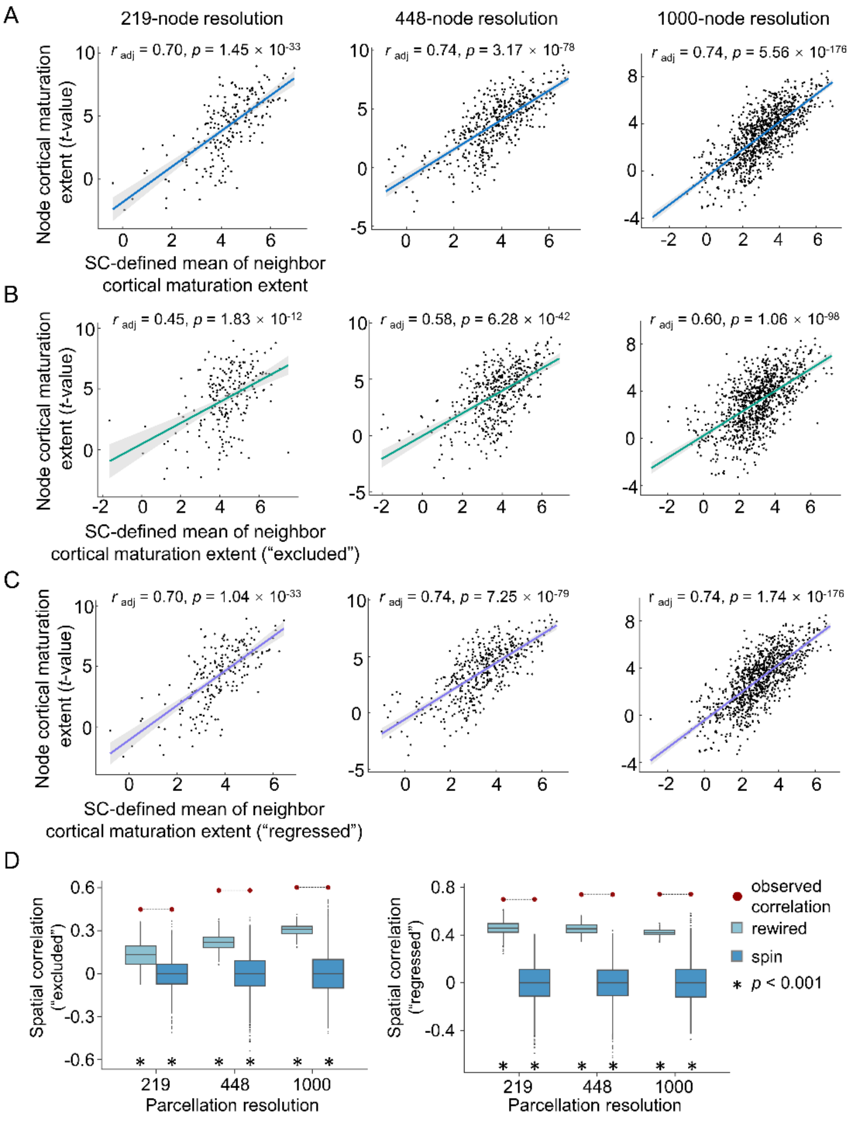

Figure S3. Associations of regional CT maturation (obtained from Statistical Model I) with the WM network architecture at three nodal resolutions. (**A**) Significant correlations were observed between the nodal CT maturation extent and the mean of its directly connected neighbors at all three nodal resolutions (two-sided). To obtain comparable correlation values under different number of observation samples, we calculated the adjusted *r*. (**B**) To determine whether these correlations were driven by the spatial proximity effect, we excluded all spatially adjoining neighbors and recalculated the mean CT maturation extent of the remaining structurally connected neighbors for each brain region ("excluded"), significant correlations were observed after re-estimating at all three nodal resolutions (two-sided). (**C**) We regressed out the effect of nodal mean Euclidean distance to its connected neighbors from the mean CT maturation extent ("regressed"), significant correlations were observed after re-estimating at all three nodal resolutions (two-sided). (**D**) The observed correlations across 3 resolutions (shown as red circles) for "excluded" (left panel) and "regressed" (right panel) analyses were compared against two baseline null models. (1) To determine whether these correlations were driven by the basic spatial embedding of the WM network, we randomly rewired edges while preserving the nodal

degree and edge length distribution of the empirical WM network ("rewired", 1000 times, shown as light blue boxes). (2) To determine whether these correlations were driven by spatial autocorrelation, we generated 1000 surrogate maps by rotating region-level cortical *t*-values ("spin test", shown as deep blue boxes). Asterisks denote statistical significance ($p < 0.001$, one-sided).

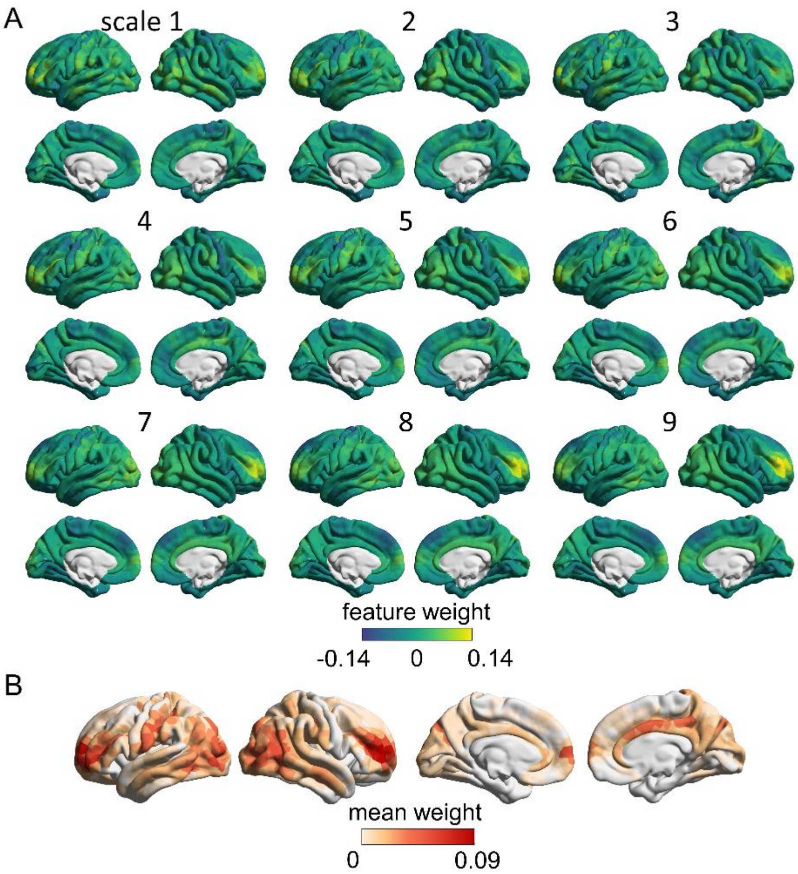

Figure S4. Feature weights for predicting CT maturation (obtained from Statistical Model I) in SVR models. (**A**) The feature weights distributions for the SVR model at 1-9 neighboring scales. (**B**) The mean weights of the nine neighboring scales, where nodes with high positive feature weights are mainly located in the frontal and parietal regions.

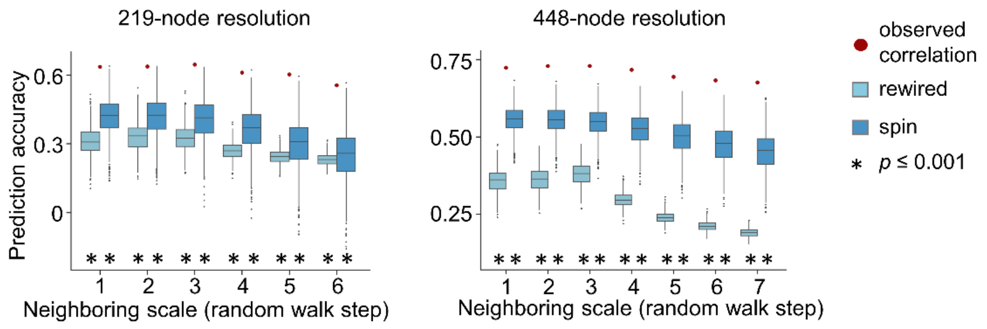

Figure S5. Validation of the network-based diffusion model at 219- and 448-nodal resolutions. Significant correlations between the predicted CT maturation and the observed CT maturation (obtained from Statistical Model I) at 219-node resolution (left panel) and 448-node resolution (right panel) by using nodal diffusive profiles at multiple neighboring scales as features in SVR model. The observed correlations (red dots) were compared to the correlations obtained from 1000 rewired tests (light blue boxes) and 1000 spin tests (deep blue boxes). Asterisks denote statistical significance ($p <= 0.001$, one-sided). See Supplementary Table S2 (219-nodal resolution) and Supplementary Table S3 (448-nodal resolution) for detailed p values.

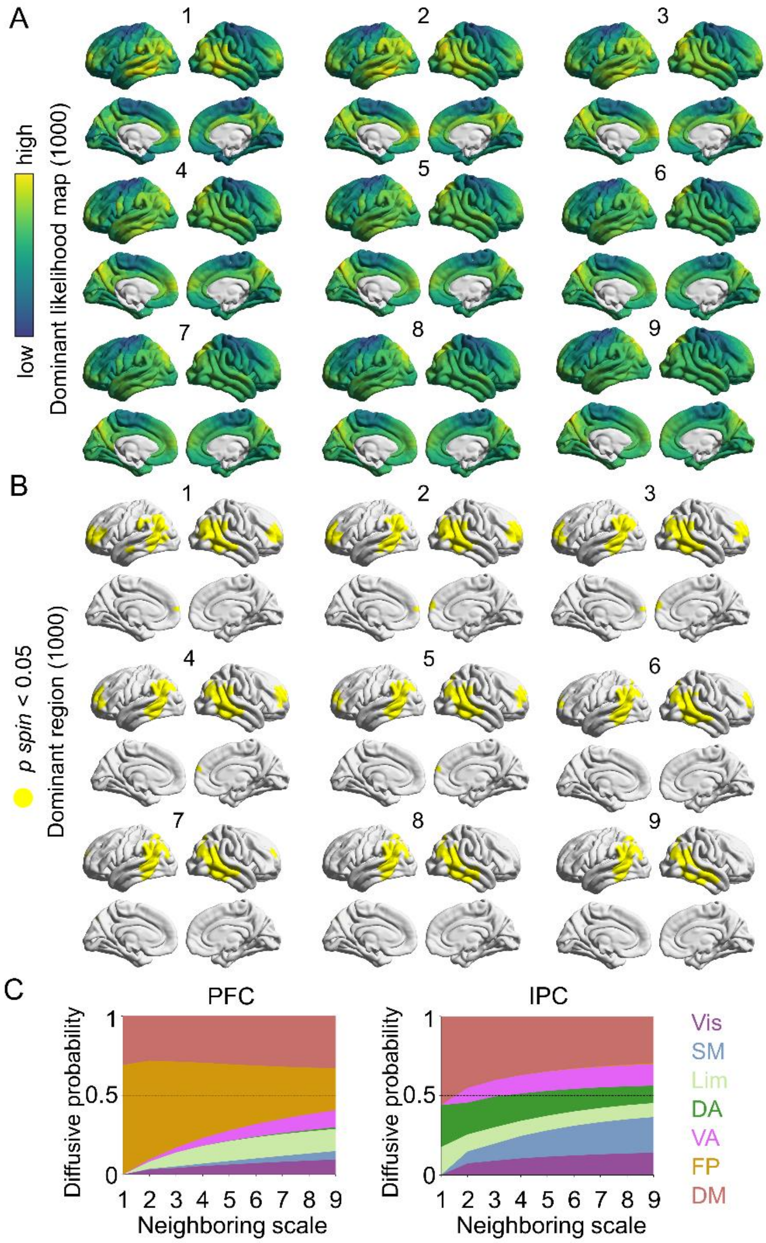

Figure S6. Dominant nodes identified by the diffusion-based approach at 1000-node resolution. (**A**) Regional distributions of dominant likelihood (cosine similarity between nodal diffusion profiles and CT maturation map (*t*-value from Statistical Model I)) across neighboring scales of 1-9 at 1000-node resolution. (**B**) Dominant region maps ($p_{spin} < 0.05$, one-sided) across 1-9 neighboring scales at 1000-node resolution. (**C**) Probability of the dominant node located in the prefrontal (left panel) and inferior parietal (right panel) cortex diffusing to each system in the 7 brain communities [23]. VIS, visual; SM, somatomotor; LIM, limbic; DA, dorsal attention; VA, ventral attention; FP, frontoparietal; DM, default mode; PFC, prefrontal cortex; IPC, inferior parietal cortex.

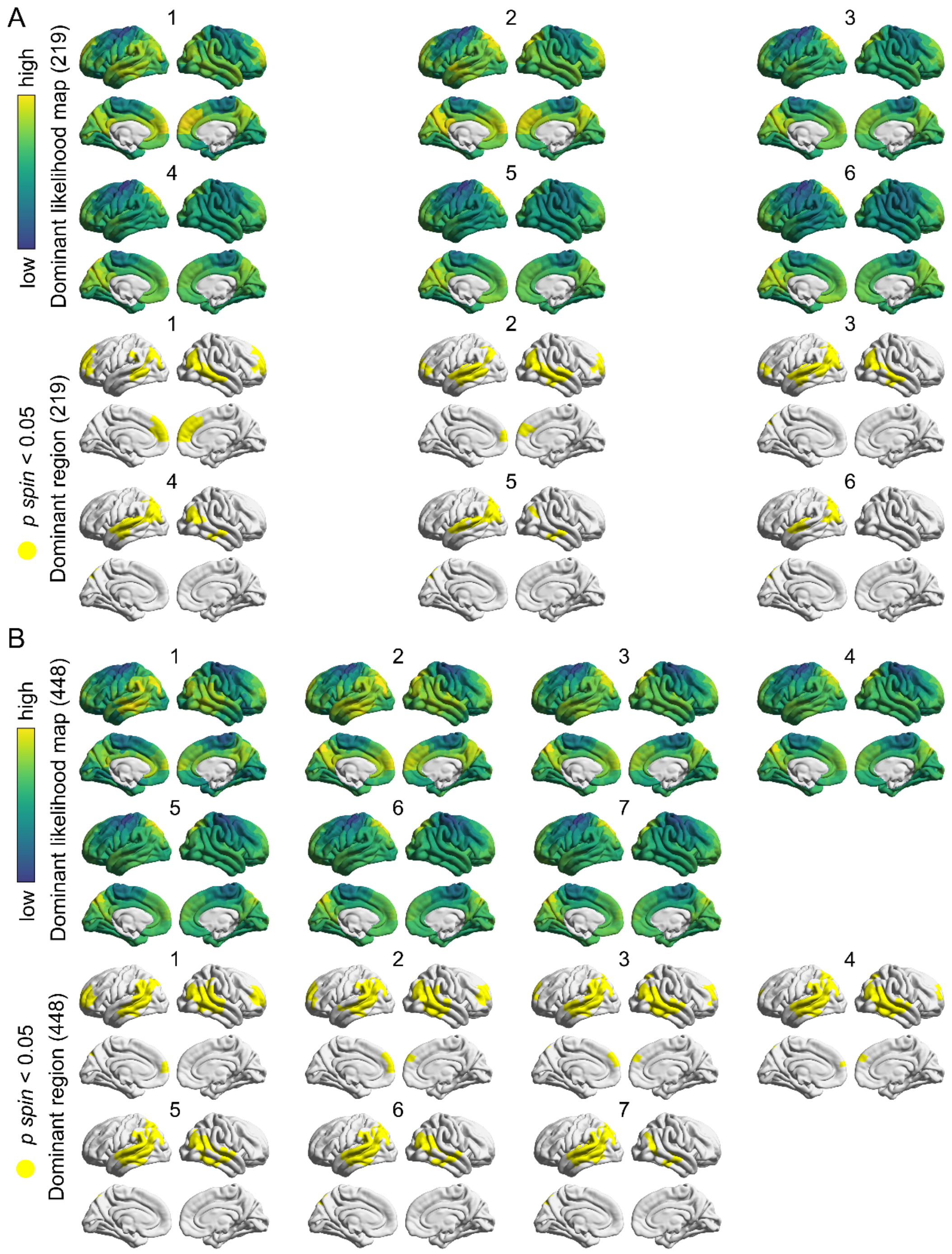

Figure S7. Validation of the dominant nodes at 219- and 448-nodal resolutions. (**A**) Dominant likelihood distribution maps (cosine similarity between nodal diffusion profiles and the CT maturation map (*t*-value from Statistical Model I)) and dominant region maps (p_{spin} < 0.05, one-sided) across neighboring scales of 1-6 at 219-nodal resolution. (**B**) Dominant likelihood distribution maps and (cosine similarity between nodal diffusion profiles and CT maturation map (*t*-value from Statistical Model I)) dominant region maps (p_{spin} < 0.05, one-sided) across neighboring scales of 1-7 at 448-nodal resolution.

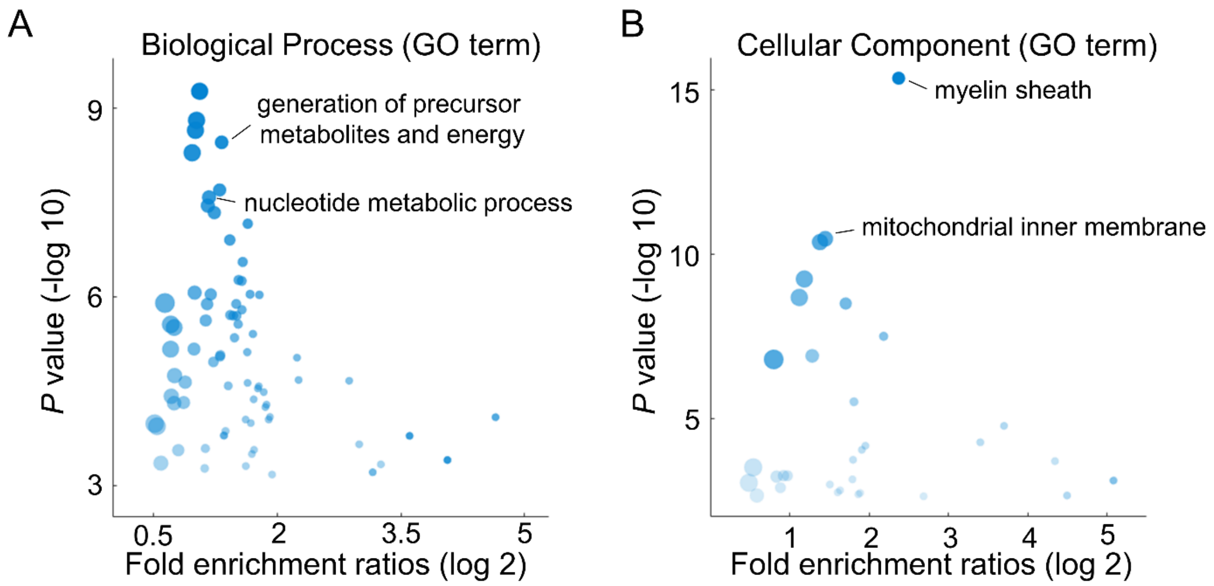

Figure S8. Gene Ontology enrichment results for negatively correlated gene sets. Volcano plot depicts Gene Ontology results for Biological Processes (**A**) and Cellular Components (**B**). The dots represent the GO terms corrected for multiple comparisons (FDR-corrected, $P < 0.05$). The size of the dot indicates the number of genes belonging to the corresponding GO term, and the transparency of the dot represents the significance of the corresponding GO term.

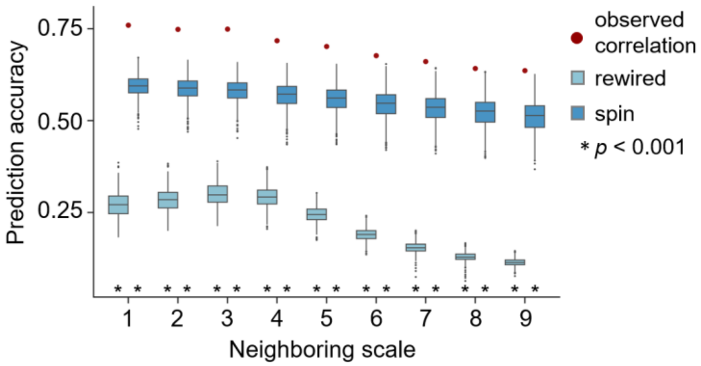

Fig. S9. Validation of the network-based diffusion model after removing ultrashort WM fibers. Significant correlations between the predicted and the observed CT maturation extent (obtained from Statistical Model I) after removing streamlines shorter than 20 mm in length from the network edges. The observed correlations (red dots) were compared to the correlations obtained from 1000 rewiring tests (light blue boxes) and 1000 spin tests (deep blue boxes). Asterisks denote statistical significance ($p < 0.001$, one-sided).

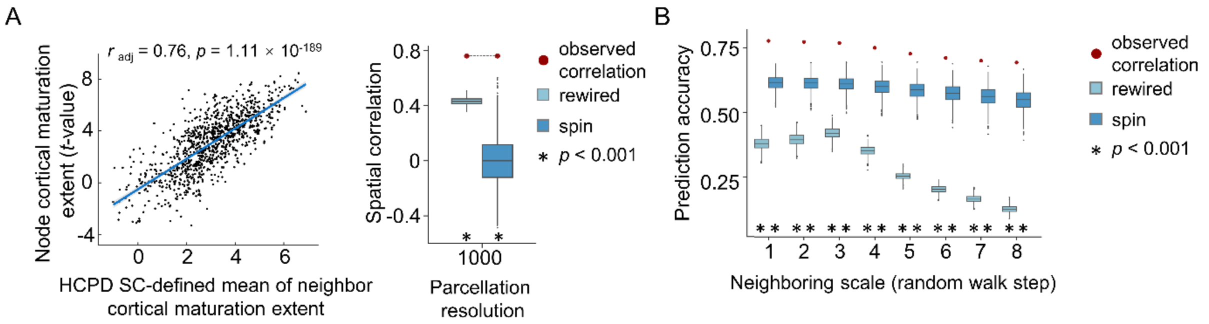

Figure S10. Validation of the WM connectome backbone effectiveness. Diffusion images with multishell diffusion gradients from replication dataset (HCP-D) were used to validate the effectiveness of the WM connectome backbone. (**A**) A significant correlation was observed between the nodal CT maturation extent (obtained from Statistical Model I) and the mean of its directly connected neighbors at 1000-node resolution (left panel, $r_{adj} = 0.76$, $P = 1.11 \times 10^{-189}$, two-sided). This observed correlation (red dots) was compared against two baseline null models (right panel, one-sided). (1) To determine whether these correlations were driven by the basic spatial embedding of the WM network, we randomly rewired edges while preserving the nodal degree and edge length distribution of the empirical WM network ("rewired", 1000 times, shown as light blue boxes). (2) To determine whether these correlations were driven by spatial autocorrelation, we generated 1000 surrogate maps by rotating region-level cortical t-values ("spin test", shown as deep blue boxes). (**B**) Significant correlations between the predicted CT maturation and the observed CT maturation by using nodal diffusive profiles at multiple neighboring scales as features in the SVR model. These observed correlations (red dots) were compared to the correlations obtained from 1000 rewired tests (light blue boxes) and 1000 spin tests (deep blue boxes). Asterisks denote statistical significance ($p < 0.001$, one-sided).

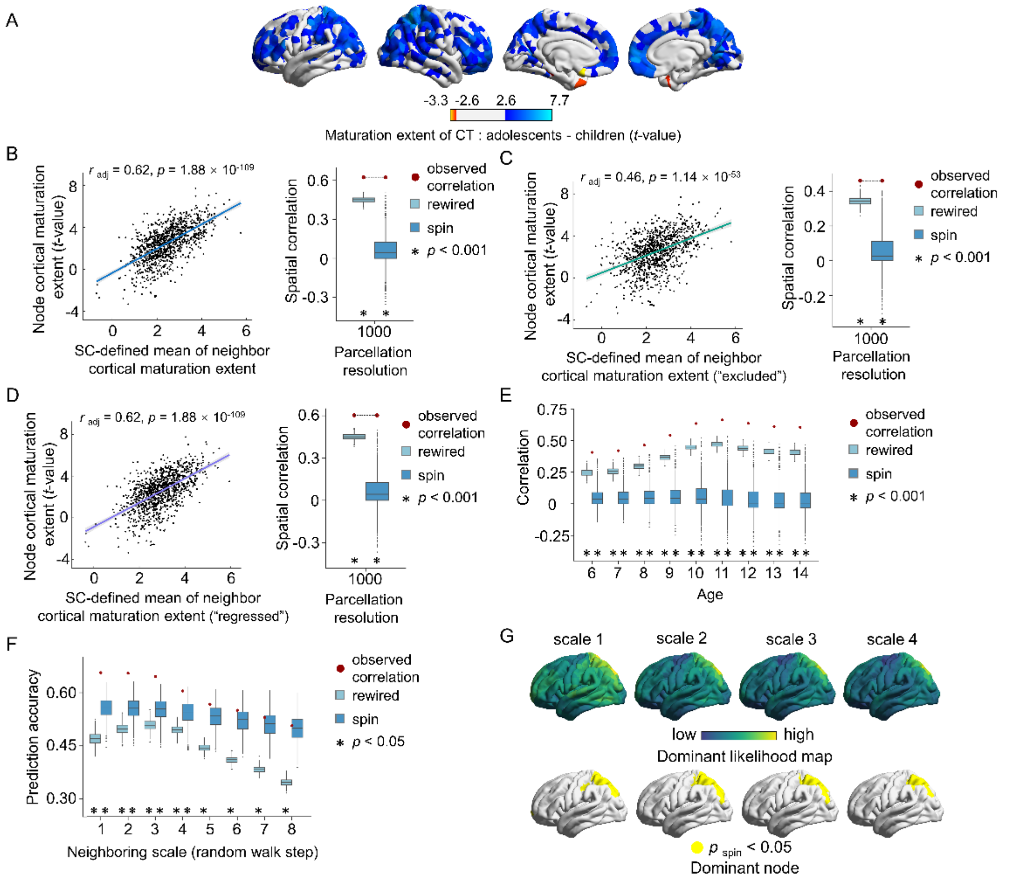

Figure S11. Validation results in the replication dataset. (**A**) Spatial maturation map of CT (obtained from Statistical Model I) from childhood to adolescence at 1000-node resolution ($P < 0.01$). A greater positive *t*-value denotes more pronounced cortical thinning with development. (**B**) Significant correlations were observed between the nodal CT maturation extent (obtained from Statistical Model I) and the mean of its directly connected neighbors (two-sided). The observed correlations were compared against two baseline null models. (1) To determine whether these correlations were driven by the basic spatial embedding of the WM network, we randomly rewired edges while preserving the nodal degree and edge length distribution of the empirical WM network ("rewired", 1000 times, shown as light blue boxes). (2) To determine whether these correlations were driven by spatial autocorrelation, we generated 1000 surrogate maps by rotating region-level cortical *t*-values ("spin test", shown as deep blue boxes). Asterisks denote statistical significance ($p < 0.001$, one-sided). Significant correlations were also observed after excluding the spatially adjoining neighbors (**C**) and regressing out the inter-node Euclidean distance (**D**). (**E**) Significant spatial correlations were observed between the nodal CT maturation rate (obtained from Statistical Model II, GAM analysis) and the mean of its directly connected neighbors at each age point. (**F**) Significant correlations between the predicted CT maturation (obtained from Statistical Model I) obtained from the SVR model using nodal diffusive profiles at multiple neighboring scales as features and the observed CT maturation. The observed correlations (red dots) were compared to the correlations obtained from 1000 rewired tests (light blue boxes) and 1000 spin tests (deep blue boxes). Asterisks denote statistical significance ($p < 0.05$, one-sided). (**G**) Dominant likelihood distribution maps and dominant region maps ($p_{spin} < 0.05$, one-sided) at 1-4 neighboring scales.

Supplementary References

1. Dong Q, Lin C. Standardized tests of the National Children's Study of China.). Beijing: Science Press (2011).

2. Somerville LH*, et al.* The Lifespan Human Connectome Project in Development: A large-scale study of brain connectivity development in 5-21 year olds. *Neuroimage* **183**, 456-468 (2018).

3. Harms MP*, et al.* Extending the Human Connectome Project across ages: Imaging protocols for the Lifespan Development and Aging projects. *Neuroimage* **183**, 972-984 (2018).

4. Yeh F-C, Wedeen VJ, Tseng W-YI. Generalized *q*-sampling imaging. *IEEE transactions on medical imaging* **29**, 1626-1635 (2010).

5. Jin Z*, et al.* Differences between generalized Q-sampling imaging and diffusion tensor imaging in visualization of crossing neural fibers in the brain. *Surg Radiol Anat* **41**, 1019-1028 (2019).

6. Yeh F-C, Verstynen TD, Wang Y, Fernández-Miranda JC, Tseng W-YI. Deterministic diffusion fiber tracking improved by quantitative anisotropy. *PloS one* **8**, e80713 (2013).

7. Tustison NJ*, et al.* N4ITK: improved N3 bias correction. *IEEE transactions on medical imaging* **29**, 1310-1320 (2010).

8. Zhao T*, et al.* Structural network maturation of the preterm human brain. *Neuroimage* **185**, 699-710 (2019).

9. Betzel RF, Griffa A, Hagmann P, Mišić B. Distance-dependent consensus thresholds for generating group-representative structural brain networks. *Network neuroscience* **3**, 475-496 (2019).

10. Hansen JY*, et al.* Local molecular and global connectomic contributions to cross-disorder cortical abnormalities. *Nature communications* **13**, 4682 (2022).

11. Bullmore E, Sporns O. Complex brain networks: graph theoretical analysis of structural and functional systems. *Nature reviews neuroscience* **10**, 186-198 (2009).

12. Miller JA*, et al.* Transcriptional landscape of the prenatal human brain. *Nature* **508**, 199-206 (2014).

13. Hansen JY, Markello RD, Vogel JW, Seidlitz J, Bzdok D, Misic B. Mapping gene transcription and neurocognition across human neocortex. *Nat Hum Behav* **5**, 1240-1250 (2021).

14. Kang HJ*, et al.* Spatio-temporal transcriptome of the human brain. *Nature* **478**, 483-489

(2011).

15. Shin J, *et al.* Cell-specific gene-expression profiles and cortical thickness in the human brain. *Cerebral Cortex* **28**, 3267-3277 (2018).

16. Richiardi J, *et al.* Correlated gene expression supports synchronous activity in brain networks. *Science* **348**, 1241-1244 (2015).

17. Vidal-Pineiro D, *et al.* Cellular correlates of cortical thinning throughout the lifespan. *Scientific reports* **10**, 21803 (2020).

18. Hawrylycz MJ, *et al.* An anatomically comprehensive atlas of the adult human brain transcriptome. *Nature* **489**, 391-399 (2012).

19. Arnatkevičiūtė A, Fulcher BD, Fornito A. A practical guide to linking brain-wide gene expression and neuroimaging data. *Neuroimage* **189**, 353-367 (2019).

20. Markello RD, Arnatkeviciute A, Poline J-B, Fulcher BD, Fornito A, Misic B. Standardizing workflows in imaging transcriptomics with the abagen toolbox. *Elife* **10**, e72129 (2021).

21. Fulcher BD, Fornito A. A transcriptional signature of hub connectivity in the mouse connectome. *Proceedings of the National Academy of Sciences* **113**, 1435-1440 (2016).

22. Chen J, Bardes EE, Aronow BJ, Jegga AG. ToppGene Suite for gene list enrichment analysis and candidate gene prioritization. *Nucleic acids research* **37**, W305-W311 (2009).

23. Yeo BT, *et al.* The organization of the human cerebral cortex estimated by intrinsic functional connectivity. *Journal of neurophysiology*, (2011).

24. Mesulam M-M. Behavioral neuroanatomy. *Principles of behavioral and cognitive neurology* **2**, 1-120 (2000).

25. Alexander-Bloch AF, *et al.* On testing for spatial correspondence between maps of human brain structure and function. *Neuroimage* **178**, 540-551 (2018).

26. Vasa F, *et al.* Adolescent Tuning of Association Cortex in Human Structural Brain Networks. *Cereb Cortex* **28**, 281-294 (2018).
