# Supplementary material for: Structural connectome architecture shapes the maturation of cortical morphology from childhood to adolescence
Source: Nat Commun. 2024 Jan 26;15:784. doi: 10.1038/s41467-024-44863-6 (PMC10817914; doi:10.1038/s41467-024-44863-6)
Supplement: Supplementary file 3 — Reporting summary [file 41467_2024_44863_MOESM3_ESM.pdf]

nature portfolio

Corresponding author(s): Yong He; Tengda Zhao

Last updated by author(s): Dec 30, 2023

Reporting Summary

Nature Portfolio wishes to improve the reproducibility of the work that we publish. This form provides structure for consistency and transparency in reporting. For further information on Nature Portfolio policies, see our Editorial Policies and the Editorial Policy Checklist.

Statistics

For all statistical analyses, confirm that the following items are present in the figure legend, table legend, main text, or Methods section.

n/a	Confirmed	
☐	☒	The exact sample size (*n*) for each experimental group/condition, given as a discrete number and unit of measurement
☐	☒	A statement on whether measurements were taken from distinct samples or whether the same sample was measured repeatedly
☐	☒	The statistical test(s) used AND whether they are one- or two-sided<br>*Only common tests should be described solely by name; describe more complex techniques in the Methods section.*
☐	☒	A description of all covariates tested
☐	☒	A description of any assumptions or corrections, such as tests of normality and adjustment for multiple comparisons
☐	☒	A full description of the statistical parameters including central tendency (e.g. means) or other basic estimates (e.g. regression coefficient) AND variation (e.g. standard deviation) or associated estimates of uncertainty (e.g. confidence intervals)
☐	☒	For null hypothesis testing, the test statistic (e.g. *F*, *t*, *r*) with confidence intervals, effect sizes, degrees of freedom and *P* value noted<br>*Give P values as exact values whenever suitable.*
☒	☐	For Bayesian analysis, information on the choice of priors and Markov chain Monte Carlo settings
☒	☐	For hierarchical and complex designs, identification of the appropriate level for tests and full reporting of outcomes
☐	☒	Estimates of effect sizes (e.g. Cohen's *d*, Pearson's *r*), indicating how they were calculated

Our web collection on statistics for biologists contains articles on many of the points above.

Software and code

Policy information about availability of computer code

Data collection	No software used
Data analysis	Diffusion images were preprocessed using MRtrix 3.0.1 (http://www.mrtrix.org/), FSL 6.0.1 (https://fsl.fmrib.ox.ac.uk/fsl/fslwiki) and ANTs 2.3.4 (https://github.com/ANTsX/ANTs).<br>Anatomical streamlines were constructed using DSI Studio 2018. Non-brain tissue removal was performed using HD-BET tool (https://github.com/NeuroAI-HD/HD-BET, Jun 16, 2021).<br>The reconstruction of cortical surface and estimation of cortical thickness were performed using FreeSurfer v6.0 (https://surfer.nmr.mgh.harvard.edu/).<br>Regional microarray expression data from Allen Human Brain Atlas were preprocessed using abagen (0.1.3) toolbox (https://github.com/rmarkello/abagen). Gene Ontology enrichment analysis was performed using online tools including ToppGene Suit (https://toppgene.cchmc.org/) and REViGO (http://revigo.irb.hr).<br>The generalized additive models were performed using the mgcv (1.8.35) R package (https://cran.r-project.org/web/packages/mgcv/index.html).<br>Support vector regression analysis was performed using LIBSVM (3.25) Matlab toolbox (https://www.csie.ntu.edu.tw/~cjlin/libsvm/).<br>Brain map visualization was implemented using BrainNet Viewer (1.7) Matlab toolbox (www.nitrc.org/projects/bnv).<br>All brain network analyses and visualization were performed using custom or public codes in Matlab 2018b (https://www.mathworks.com/products/matlab.html), Python 3.7.12 (https://www.python.org), and R 3.6.3 (https://www.r-project.org).<br>All analysis codes used in this study are publicly available at https://github.com/Xinyuan-Liang/SC-shapes-the-maturation-of-cortical-morphology

For manuscripts utilizing custom algorithms or software that are central to the research but not yet described in published literature, software must be made available to editors and reviewers. We strongly encourage code deposition in a community repository (e.g. GitHub). See the Nature Portfolio guidelines for submitting code & software for further information.

Data

Policy information about availability of data

All manuscripts must include a data availability statement. This statement should provide the following information, where applicable:

- Accession codes, unique identifiers, or web links for publicly available datasets
- A description of any restrictions on data availability
- For clinical datasets or third party data, please ensure that the statement adheres to our policy

For the Discovery Dataset (CBD dataset) and the Replication Dataset (HCP-D), all data required for reproducing our findings have been publicly available, including the individual regional cortical thickness matrices, structural connectivity matrices, the intermediate results during analysis, and the data for visualizing main figures. They are stored in a publicly accessible cloud repository (https://github.com/Xinyuan-Liang/SC-shapes-the-maturation-of-cortical-morphology).
For the Discovery Dataset (CBD dataset), the raw neuroimaging data used in this study are available upon request from the corresponding authors.
For the Replication Dataset (HCP-D), the raw image scans are publicly available at https://nda.nih.gov/.
The BrainSpan Atlas dataset is publicly available at http://brainspan.org/static/download.html.
The AHBA dataset is publicly available at https://human.brain-map.org/static/download.

Research involving human participants, their data, or biological material

Policy information about studies with human participants or human data. See also policy information about sex, gender (identity/presentation), and sexual orientation and race, ethnicity and racism.

Reporting on sex and gender	Participants' sex (on basis of self-report) was used for descriptive purposes and as a covariate in analyses. Structural connectomes and cortical measures were generated from participants of both sexes.
Reporting on race, ethnicity, or other socially relevant groupings	Race, ethnicity and other socially relevant information were not included in this study.
Population characteristics	The Discovery Dataset (CBD) included a longitudinal cohort of 358 typically developing participants (aged 6-14 years, 651 scans from 195 males and 163 females).<br>The Replication Dataset (HCP-D) included 301 typically developing participants aged 5 to 14 years (183 females).
Recruitment	CBD: All children were recruited from primary schools in Beijing and were cognitively normal, assessed by a well-validated Chinese standardized cognitive ability test, significant physical illness or history of neurological/psychiatric disorders, significant head injuries, abuse of illegal drugs and alcohol, or contraindications for MRI.<br>HCP-D: Participants in this sample were recruited across four sites: Harvard University, University of California-Los Angeles, University of Minnesota, and Washington University in St. Louis. Exclusion. Participants are healthy individuals aged 5-21 years. Exclusion criteria for recruitment included (1) premature birth (< 37weeks gestation); (2) serious neurologic condition (3) serious endocrine condition; (4) long-term use of immunosuppressants or steroids; (5) history of serious head injury; (6) hospitalization >2d for certain physical or psychiatric conditions or substance use; (7) treatment >12 months for psychiatric conditions; (8) claustrophobia; or (9) pregnancy or other contraindications for MRI.
Ethics oversight	CBD dataset: Informed written consent was obtained from all participants and at least one parent/guardian. All procedures were approved by the Ethics Committee of Beijing Normal University at Beijing Normal University, Beijing, P. R. China.<br>HCP-D dataset: All procedures were approved by a central Institutional Review Board administered at Washington University in St. Louis.

Note that full information on the approval of the study protocol must also be provided in the manuscript.

Field-specific reporting

Please select the one below that is the best fit for your research. If you are not sure, read the appropriate sections before making your selection.

☒ Life sciences ☐ Behavioural & social sciences ☐ Ecological, evolutionary & environmental sciences

For a reference copy of the document with all sections, see nature.com/documents/nr-reporting-summary-flat.pdf

Life sciences study design

All studies must disclose on these points even when the disclosure is negative.

Sample size	The Discovery Dataset (CBD) included a longitudinal cohort of 358 typically developing participants (aged 6-14 years, 651 scans from 195 males and 163 females). For the Replication Dataset (HCP-D), we included 301 typically developing participants aged 5 to 14 years (183 females). The sample size was determined based on the availability of brain scans in these two datasets and our sample size is similar to those reported in previous publications. We validated the reproducibility of the results using the two independent datasets, although no sample size calculation was performed.<br>For the Allen Human Brain datasets (https://human.brain-map.org/static/download) and BrainSpan datasets (http://brainspan.org/static/download.html), the sample sizes are consistent with the publicly available original data.

Data exclusions	For the Discovery Dataset (CBD), a total of 130 scans were excluded. Specifically, 76 scans were excluded due to artifacts in T1-weighted (T1w) images, 39 scans were excluded due to high in-scanner motion (maximum head motion > 3 mm) and 15 scans excluded due to serious signal dropout, respectively in diffusion MRI (dMRI) images. Finally, 521 scans from 314 participants (aged 6-14 years, 153 females) were included in the study.<br>For the Replication Dataset (HCP-D), we first excluded 3 participants with anatomical anomalies and 17 participants with notable myelin map quality issues according to each subject's quality control report of structural MR images. After these, T1w images of 98 children (5-10 years, mean age 8.72 ± 0.99 years, 32 males) and 203 adolescents (10-14 years, mean age 12.17 ± 1.28 years, 86 males) were eventually included.
Replication	Our analysis was conducted across two independent datasets and three cortical parcellation resolutions. We report cortical thinning patterns primarily located in lateral frontal and parietal heteromodal nodes during childhood and adolescence, which is structurally constrained by white matter network architecture and is particularly represented using a network-based diffusion model. These results are largely consistent across three cortical parcellations and are highly reproducible across two independent datasets.
Randomization	No randomization was performed as this study does not include experimental groups.
Blinding	Blinding is not relevant to this study because it does not include experimental groups.

Reporting for specific materials, systems and methods

We require information from authors about some types of materials, experimental systems and methods used in many studies. Here, indicate whether each material, system or method listed is relevant to your study. If you are not sure if a list item applies to your research, read the appropriate section before selecting a response.

Materials & experimental systems

n/a	Involved in the study
☒	☐ Antibodies
☒	☐ Eukaryotic cell lines
☒	☐ Palaeontology and archaeology
☒	☐ Animals and other organisms
☒	☐ Clinical data
☒	☐ Dual use research of concern
☒	☐ Plants

Methods

n/a	Involved in the study
☒	☐ ChIP-seq
☒	☐ Flow cytometry
☐	☒ MRI-based neuroimaging

Magnetic resonance imaging

Experimental design

Design type	Structural MRI, diffusion-weighted MRI
Design specifications	No trials
Behavioral performance measures	No behavioral measures

Acquisition

Imaging type(s)	Structural , diffusion
Field strength	3T
Sequence & imaging parameters	For the Discovery Dataset (CBD), high-resolution T1w images for each subject were scanned at Peking University using a 3T Siemens Prisma scanner. T1w images were acquired using the following parameters: repetition time (TR) = 2530 ms, echo time (TE) = 2.98 ms, inversion time (TI) = 1100 ms, flip angle (FA) =7 ∘, acquisition matrix = 256×224, field of view (FOV) = 256×224 mm2, slice number = 192, in-plane resolution = 1.0 × 1.0 mm, slice thickness = 1 mm, bandwidth (BW) = 240 Hz/Px. Diffusion-weighted images were acquired using the high angular resolution diffusion imaging (HARDI) sequence with a 64-channel head coil with parameters as follows: TR = 7500 ms, TE = 64 ms, acquisition matrix = 112×112, FOV = 224×224 mm2, slices = 70, in-plane resolution = 2.0 × 2.0 mm, slice thickness = 2.0 mm, BW = 2030 Hz/Px, phase encoding = P->A, 64 diffusion weighted directions (b-value = 1000 s/mm2) with 10 non-diffusion weighted b0 (0 s/mm2). Meanwhile, additional fieldmap images were obtained for EPI distortion correction with the following parameters: acquisition matrix = 112×112, FOV =224×224 mm2, slices = 70, slice thickness = 2.0 mm, TR = 695 ms, TE1 = 4.92 ms, TE2 = 7.38 ms, in-plane resolution = 2.0 × 2.0 mm.<br>For the Replication Dataset (HCP-D), high-resolution T1w images were scanned on a 3T Siemens Prisma using the following parameters 5: TR = 2500 ms, TE = 1.8/3.6/5.4/7.2 ms, TI = 1000 ms, FA = 8°, in-plane resolution = 0.8 × 0.8 mm, slice thickness = 0.8 mm, and in-plane acceleration factor = 2. Parameters for diffusion MRI were as follows: TR = 3230 ms, TE = 89 ms, in-plane resolution = 1.5 × 1.5 mm, slice thickness = 1.5 mm, multiband acceleration factor = 4, 92-93 directions per shell (b = 1500/3000 mm2). There are 28 b0 volumes equally interspersed across four consecutive dMRI runs. Therefore, each individual acquired two images with 199 volumes in the opposite phase encoding direction

(AP and PA).

Area of acquisition | Whole brain

Diffusion MRI ☒ Used ☐ Not used

Parameters: CBD: 1 shell (b vals: 1000), 64 directions, 10 non-diffusion weighted b0. HCP-D: 3 shells (b vals: 1500, 3000), 92-93 directions per shell, 28 b0 volumes equally interspersed across four consecutive dMRI runs.

Preprocessing

Preprocessing software	Diffusion data was preprocessed using MRtrix 3.0.1, FSL 6.0.1 and ANTs 2.3.4. Regional microarray expression data from Allen Human Brain Atlas were preprocessed using abagen (0.1.3) toolbox.
Normalization	For the white matter network construction, the deterministic fiber tracking and cortical reconstruction were performed in the individual space. The cortical parcellation for each participant was linearly transformed from the native T1w space into the native dMRI space using affine transformation with the individual mean b0 image as the co-registered target. For the abagen workflow, the MNI coordinates of the tissue samples were updated to those generated via nonlinear registration using the ANTs.
Normalization template	We constructed a custom surface template by averaging the cortical surfaces of all available participants using the FreeSurfer pipeline. The atlas in the standard fsaverage space was initially transformed to this custom template and subsequently warped into each participant's native surface space to obtain corresponding regional cortical thickness measurements. To obtain regional gene expression values, tissue samples were assigned to the brain regions in MNI152 space using the abagen toolbox.
Noise and artifact removal	CBD: Diffusion data were first denoised, and Gibbs ringing artifacts were removed using MRtrix 3.0.1. Next, we corrected eddy current-induced distortions, head movements, and signal dropout using the FSL eddy tool. Then, to make the susceptibility-induced EPI distortion correction, we fed the eddy-corrected DW images and corresponding fieldmap images into the FUGUE tool (https://fsl.fmrib.ox.ac.uk/fsl/fslwiki/FUGUE/Guide#Making_Fieldmap_Images_for_FEAT) to remove EPI susceptibility artifacts. Finally, B1 field inhomogeneity was corrected for the dMRI images with the N4 algorithm available in ANTs.<br>HCP-D: Diffusion data were first denoised, and Gibbs ringing artifacts were removed. Then, we used topup/eddy to correct the EPI distortions, eddy currents, subject movement distortions, and signal dropout. Finally, B1 field inhomogeneity was corrected for the dMRI images.
Volume censoring	All volumes were visually inspected.

Statistical modeling & inference

Model type and settings	Mass univariate and predictive. To estimate the maturation of cortical thickness from childhood to adolescence, we applied a mixed linear analysis with the sex term included as the covariate and the group term as the main effect for each brain node. We trained a support vector regression model with diffusive profiles at all neighboring scales of a brain node as input features to predict its nodal CT maturation extent. Reported spatial correlations accounted for spatial autocorrelation by utilizing spin-test null permutation models.
Effect(s) tested	We tested whether the spatial correlation was lower or higher than the correlations obtained using two baseline null models (spin test and rewired test).

Specify type of analysis: ☒ Whole brain ☐ ROI-based ☐ Both

Statistic type for inference<br>(See Eklund et al. 2016)	Analyses considered all nodal parcellation.
Correction	False discovery rate (FDR), Bonferroni

Models & analysis

n/a	Involved in the study
☒	☐ Functional and/or effective connectivity
☐	☒ Graph analysis
☐	☒ Multivariate modeling or predictive analysis

Graph analysis	Group-level and individual-level binary white matter networks. The binary group-level WM backbones were generated using a consensus approach that preserves the connection length distributions of individual white matter networks in the children group. The diffusive probabilities of the white matter network were estimated using a random walk modeling approach.
Multivariate modeling and predictive analysis	We trained a support vector regression model with diffusive profiles at all neighboring scales of a brain node as input features to predict its nodal CT maturation extent. We performed principal component analysis on

the gene expression matrix to calculate the first principal component score of each gene set's transcription level in dominant and non-dominant regions.
